# Traumatic dental injuries in preschool‐age children: Prevalence and risk factors

**DOI:** 10.1002/cre2.165

**Published:** 2019-01-30

**Authors:** Catherine D. Born, Tate H. Jackson, Lorne D. Koroluk, Kimon Divaris

**Affiliations:** ^1^ Department of Orthodontics, School of Dentistry University of North Carolina‐Chapel Hill Chapel Hill North Carolina USA; ^2^ Department of Pediatric Dentistry, School of Dentistry University of North Carolina‐Chapel Hill Chapel Hill North Carolina USA; ^3^ Department of Epidemiology, Gillings School of Global Public Health University of North Carolina‐Chapel Hill Chapel Hill North Carolina USA

**Keywords:** children, clinical predictors, risk, trauma

## Abstract

This study examined the prevalence, socio‐demographic correlates, and clinical predictors of traumatic dental injuries (TDIs) in the primary dentition among a community‐based sample of preschool‐age children. The sample comprised 1,546 preschool‐age children (mean age 49 [range: 24–71] months) in North Carolina public preschools, enrolled in a population‐based investigation among young children and their parents in North Carolina. Information on socio‐demographic, extraoral, and intraoral characteristics was collected and analyzed with bivariate and multivariate methods, including logistic regression modeling and marginal effects estimation. The prevalence of dental trauma was 47% and 8% of TDI cases were “severe” (pulp exposure, tooth displacement, discolored or necrotic tooth, or tooth loss). In bivariate analyses, overjet and lip incompetence were significantly associated with TDI. Overjet remained positively associated with severe trauma in multivariate analysis, *OR* = 1.4, 95% confidence interval (CI) [1.2, 1.6], corresponding to an absolute 1.3%, 95% CI [0.7, 1.8], increase in the likelihood of severe trauma, per millimeter of overjet. Children with increased overjet (>3 mm) were 3.8, 95% CI [2.0, 7.4], times as likely to have experienced severe TDI compared with those with ≤3 mm. Overjet is a strong risk factor for TDIs in the primary dentition. Incorporating and operationalizing this information may help TDI prevention and related anticipatory guidance for families of preschool‐age children.

## 1 INTRODUCTION

Traumatic dental injuries (TDIs) are relatively common among children (World Health Organization, 2019). It is estimated that 17–50% of adolescents and adults experience dental trauma to one or more permanent teeth (Goslee, Lee, Rozier, & Quinonez, 2006; Kaste, Gift, Bhat, & Swango, 1996; Shulman & Peterson, 2004) and 9–40% of children experience trauma in their primary dentition (Andreasen & Ravn, 1972; Bonini, Bönecker, Braga, & Mendes, 2012; Feldens, Kramer, Ferreira, Spiguel, & Marquezan, 2010; Goettems et al., 2012; Jones, Mourino, & Bowden, 1993; Norton & O'Connell, 2012; Oliveira, Marcenes, Ardenghi, Sheiham, & Bonecker, 2007; Piovesan, Guedes, Casagrande, & Ardenghi, 2012; Wendt et al., 2010; Table 1). The wide range in reported prevalence of TDIs in the primary dentition is likely due to variation in the studied populations and sample characteristics, study design, and injury diagnosis and classification (Bastone, Freer, & McNamara, 2000). The clinical consequences of TDIs to the primary dentition are obvious and measureable; however, there are also potential sequelae to the developing succedaneous teeth including hypoplastic defects, root dilacerations, and other enamel or developmental disturbances that are not seen until months or years after the injury when the permanent successors erupt (Andreasen, Sundström, & Ravn, 1971; Lenzi, Alexandria, Ferreira, & Maia, 2015). Overall, consequences of TDIs extend well beyond the traditional clinical implications and can affect the quality of life of those affected and their families. Negative economic, social, and psychological impacts due to TDI have been well documented (Borum & Andreasen, 2001; Fakhruddin, Lawrence, Kenny, & Locker, 2008; Lee & Divaris, 2009; Nguyen, Kenny, & Barrett, 2004), highlighting the public health problem posed by injury to the teeth, face, and jaws.

**Table 1:** Prevalence of traumatic dental injury in the primary dentition, classification of increased overjet, and estimates of association between trauma and increased overjet

Author	Year	Country	Sample size	Age range (months)	Prevalence TDI (%)	Proportion enamel only (%)	Proportion enamel and dentin (%)	Increased overjet (mm)	Odds ratio for increased overjet	Prevalence ratio for increased overjet
Andreasen & Ravn, 1972	1972	Denmark	487	36–95	30					
Jones et al., 1993	1993	United States	493	36–59	23					
Oliveira et al., 2007	2007	Brazil	892	5–59	9.4	68.8	13.8			
Feldens et al., 2010	2010	Brazil	888	36–71	36.4			>2	1.86 (1.39–2.50)	1.50 (1.23–1.83)
Goettems et al., 2012	2010	Brazil	501	24–71	40			≥3		
Wendt et al., 2010	2010	Brazil	571	12–71	36.6					
Bonini et al., 2012	2012	Brazil	376	36–59	27.7	58.4	17.6	>3		1.74 (1.25–2.41)
Norton & O′Connell, 2012	2012	Ireland	839	9–84	25.6	39.4		3.5–6	1.15 (0.83–1.59)	
								>6	2.99 (2.0–4.47)	
Piovesan et al., 2012	2012	Brazil	441	12–59	31.7	86.9	4.2	>3		1.90 (1.34–2.70)

*Note*. Summary of past studies. TDI: traumatic dental injury.

The high prevalence of TDIs and their negative impact on quality of life have motivated research into possible etiologic factors. It is common ground that dental trauma etiology is multifactorial and complex. In 2009, Glendor suggested that the three main etiologic factors for TDIs can be grouped in the domains of “human behavior,” which generally includes risk‐taking behaviors, conditions such as attention‐deficit/hyperactivity disorder, and others; “environmental determinants,” wherein more contextual parameters, such as material deprivation, or an “unsafe” environment are included; and “oral factors,” including increased overjet with protrusion, lip incompetence, and other intraoral and extraoral factors (Glendor, 2009). This triad is certainly not an all‐inclusive list but offers a helpful categorization of all postulated risk factors for dental trauma. Additional risk factors that do not necessarily fall into one of these three categories but might also increase the risk of TDIs are body mass index (BMI), sex, presence of illness, learning difficulties, physical limitations, inappropriate use of teeth, and oral piercings (Zaleckiene, Peciuliene, Brukiene, & Drukteinis, 2014).

Although previous studies have investigated the prevalence of TDIs and the association of oral factors and other characteristics such as sex, BMI, and nonnutritive sucking habits (Andreasen & Ravn, 1972; Bonini et al., 2012; Feldens et al., 2010; Martins et al., 2014; Norton & O'Connell, 2012; Piovesan et al., 2012; Soriano, Caldas, De Carvalho, & Amorim Filho, 2007), very few studies have examined TDI in the primary dentition in the United States (Jones et al., 1993), and none has actually incorporated this information in a clinically useful risk model. Such a tool could be used for risk assessment and would be beneficial for family education, screenings, personalized prevention, risk reduction, and planning early orthodontic treatment. The present study aimed to address this gap and sought to (a) examine the prevalence of TDIs in the primary dentition among a community‐based cohort of preschool‐age children, (b) determine the socio‐demographic and clinical predictors of TDIs in this population, and (c) use this information to develop a risk model for TDIs.

## 2 MATERIALS AND METHODS

### 2.1 Study population

The sample was drawn from the Zero‐Out Early (ZOE) Childhood Caries study, a prospective and population‐based investigation among young children and their parents in North Carolina (NC). The sample comprised three contiguous “waves”: ZOE 1.0 (*n* = 345, conducted among Early Head Start [EHS] during 2012–2013 and preliminary results reported by Born, Divaris, Hom, & Rozier, 2015), ZOE‐pilot (*n* = 353, conducted among Head Start [HS] during 2013–2014), and ZOE 2.0 (in progress; first 848 participants included in this analysis). All phases of the study were undertaken with an identical clinical examination protocol and received institutional review board approval (University of North Carolina, Office of Human Research Ethics #08‐1185 and #14‐1992). The study design and patient selection are described in detail elsewhere (Born et al., 2015; Ginnis et al., 2019).

The participants in ZOE comprise a multiethnic cohort of preschool‐age children in NC, with African American and Hispanic children being the most represented racial/ethnic groups, and between the ages of three and four. Children were from low‐income families and were either enrolled in EHS/HS or were Medicaid‐enrolled controls (in ZOE 1.0; Born et al., 2015). Selection of HS programs and centers in ZOE was based upon a representative sample design (probability proportional to HS center size) of all HS (total enrollment in 2017 was about 17,000) in NC. To be included in the study, children had to be enrolled and have undergone a clinical examination as part of ZOE 1.0, ZOE‐pilot, or ZOE 2.0 study waves. Children were excluded from the present analyses if they were <24 months or >71 months of age or had key socio‐demographic (e.g., gender) or clinical (e.g., trauma) information missing.

### 2.2 Data collection

The clinical exams in all ZOE phases followed a previously described standardized protocol (Ginnis et al., 2019) and were performed in EHS/HS centers during normal school hours. In brief, examination teams (three across the state of NC, in ZOE 2.0 Divaris & Joshi, 2018, including seven clinical examiners) used portable equipment to conduct clinical examinations under field conditions. The examination was performed in the following sequence. (a) Height and weight were obtained after removing heavy clothing and shoes. (b) The child was accompanied to the dental chair by the recorder while BMI and BMI percentile for age and sex were calculated using a tablet application; (c) the examiner brushed the child's teeth. (d) A clinical examination was done to record tooth‐surface conditions including dental trauma using a modified Ellis classification criteria (Ellis & Davey, 1970), on the most‐affected upper anterior tooth (if more than one), as follows: simple enamel‐only fracture, extensive fracture with dentin and no pulp involvement, traumatic pulp exposure, tooth displacement, necrotic/discolored tooth, and total tooth loss due to trauma. The Ellis' modified classification system provides an anatomical and numerical basis for classification with a hierarchical structure that groups various injuries into categories (Bastone et al., 2000; Feliciano & de França Caldas, 2006; Pagadala & Tadikonda, 2015). Additional information was systematically collected on extraoral (e.g., profile and lip competence) and intraoral (e.g., overjet, overbite, molar, and canine classification) clinical parameters, as well as behavior using the Frankl scale (American Academy of Pediatric Dentistry Clinical Affairs Committee – Behavior Management Subcommittee, 2015).

Profile was classified as convex, straight, or concave based on the angle between soft tissue nasion, soft tissue A point, and soft tissue B point. Lips were considered incompetent if the lips were everted and separated by ≥3 mm. To assess intraoral characteristics, children were asked to bite down on their back teeth; frequently, children were instructed to say “cheese” or swallow to aid in assessment of occlusion. The examiner then made an effort to guide the child into centric relation (Ginnis et al., 2019). Overjet was measured using a periodontal probe from the incisal edge of the most anteriorly placed maxillary central incisor to the labial portion of the most lingually placed mandibular central incisor. Overbite was assessed as the amount of vertical overlap of the maxillary central incisors over the mandibular central incisors and was reported as a percentage of the total height of the mandibular incisor. Both right and left molar and canine relationships were reported and were categorized as Classes I, II, or III. Children with an edge‐to‐edge molar relationship were classified as Class II. Similarly, children did not have to be a full cusp Class III in order to be classified as Class III. Socio‐demographic (e.g., race/ethnicity and sex) information was collected from the participating families via a self‐administered parent questionnaire that was digitized using a Teleform® (scan) system.

### 2.3 Analytical approach

#### 2.3.1 Statistical analysis

Data were initially analyzed using descriptive methods and univariate statistics (e.g., mean, standard deviation, median, and range). Bivariate tests of association between severe TDI prevalence (binary definition: no trauma, simple crown fracture, extensive crown fracture without pulp involvement vs. extensive fracture with pulp involvement, tooth displacement, necrotic discolored tooth, or total tooth loss due to trauma) included χ^2^ tests, Fisher's exact tests, analysis of variance, or *t* tests, and pairwise correlations using a conventional *P* < 0.05 statistical significance threshold. A Šidák correction was applied to account for multiple testing in pairwise correlations. Because severe TDI was not a common occurrence (<20%), the use of logistic (vs. log binomial) regression for multivariate modeling was justified. Selection of covariates for inclusion in the final multivariate model departed from a “full” model including all variables associated with TDIs in bivariate analysis and employed a backward variable elimination criterion using a likelihood ratio χ^2^ test (*P* < 0.20) comparing the fit of “full” versus “reduced” models. To facilitate interpretation and determination of clinical relevance, we estimated average marginal effects (i.e., changes in the predicted probability [in percentage points, p.p.] of having severe TDI adjusting for all other model covariates) and 95% confidence intervals (CI). We based our inference on adjusted marginal effect estimation (model‐predicted probabilities) and 95% CIs. We examined the predictive properties of the final model via conventional classification metrics (e.g., sensitivity and specificity), proportion of subjects correctly classified, and receiver operating characteristic area under the curve. All analyses were done with Stata (StataCorp LLC, College Station, TX) version 15.1.

## 3 RESULTS

**Figure 1:**
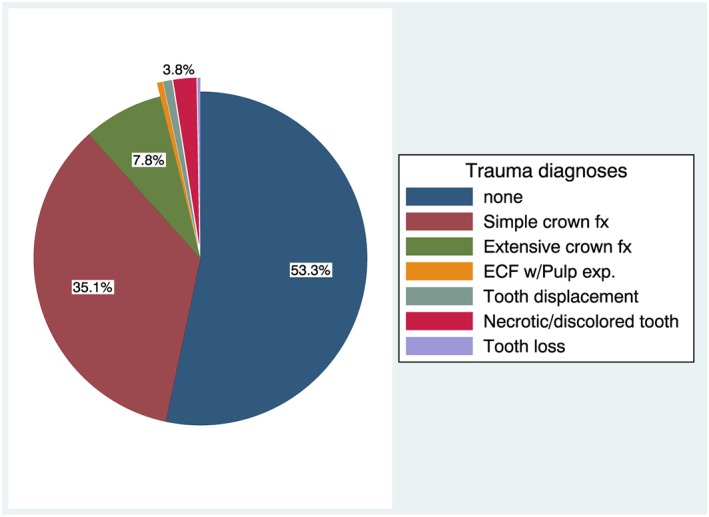
Distribution of traumatic dental injury diagnoses in the study sample

The study population included 1,546 preschool‐age children (mean age 49 [range: 24–71] months): 770 (50%) were male and 776 (50%) were female. The prevalence of dental trauma was 47%. Three quarters of TDI cases had enamel‐only fractures, whereas a small proportion (12%) showed evidence of more extensive trauma (dentin involvement or worse). The prevalence and distribution of dental trauma diagnoses are presented in Figure 1.

**Figure 2:**
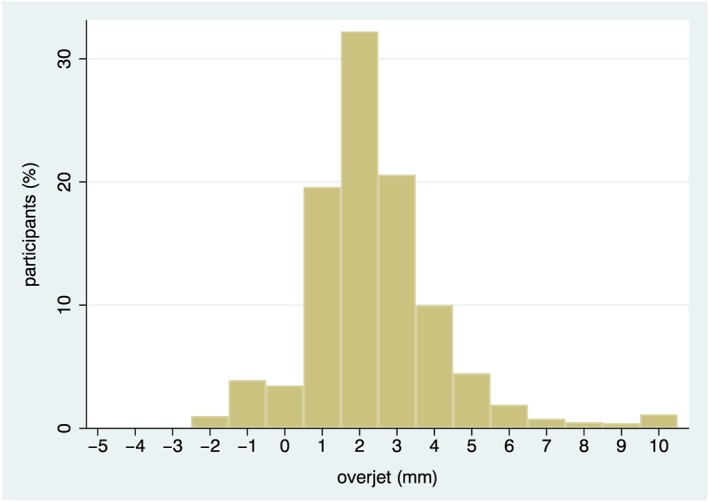
Distribution of overjet values (mm)

The socio‐demographic, intraoral, and extraoral characteristics of study participants, overall and stratified by incidence of severe dental injury are presented in Table 2. In bivariate analyses, lip incompetence and overjet (distribution of values shown in Figure 2) were significantly associated with TDI (*P* < 0.05), whereas age, BMI, and canine occlusion showed weaker positive associations. The pairwise correlation coefficients between severe trauma and overjet and lip competence were 0.14 and −0.09, respectively, with *P* < 0.05 after a Šidák correction for multiple testing.

**Table 2:** Descriptive information of participating children and their association with severe traumatic dental injury

Children's characteristics	All participants		Severe traumatic dental injury				*P* value
			No		Yes		
	*N* or mean	Column (%) or *SD*	*N* or mean	Row (%) or *SD*	*N* or mean	Row (%) or *SD*	χ^2^, Fisher's exact, or *t* test
Entire sample	1,546	100.0	1,488	96.3	58	3.8	
Sex							0.271
Male	770	49.8	737	95.7	33	4.3	
Female	776	50.2	751	96.8	25	3.2	
Age (years)							0.069
2	94	6.1	93	98.9	1	1.1	
3	554	35.8	530	95.7	24	4.3	
4	618	40.0	601	97.3	17	2.8	
5	280	18.1	264	94.3	16	5.7	
Continuous (months)	49.5	9.4	49.5	9.4	50.7	10.1	0.319
Body mass index (BMI)							0.161
Underweight	144	9.6	142	98.6	2	1.4	
Normal	986	66.0	943	95.6	43	4.4	
Overweight	202	13.5	198	98.0	4	2.0	
Obese	162	10.8	155	95.7	7	4.3	
*Missing*	52						
Frankl score							0.657
1	55	3.6	52	94.6	3	5.5	
2	118	7.6	114	96.6	4	3.4	
3	268	17.3	261	97.4	7	2.6	
4	1,105	71.5	1,061	96.0	44	4.0	
Overjet							<0.005
4 mm or more	271	19.2	250	92.3	21	7.8	
<4 mm	1,138	80.8	1,106	97.2	32	2.8	
*Missing*	137						
Continuous (mean, *SD*)	2.4	1.8	2.3	1.8	3.7	2.3	<0.005
Overbite (%)							0.542
Negative	63	4.5	61	96.8	2	3.2	
0 to <25	371	26.6	358	96.5	13	3.5	
25 to <50	258	18.5	245	95.0	13	5.0	
50 to <75	440	31.6	428	97.3	12	2.7	
75 to 100	262	18.8	250	95.4	12	4.6	
*Missing*	152						
Profile							0.429
Convex	1,417	92.9	1,362	96.1	55	3.9	
Not convex	109	7.1	107	98.2	2	1.8	
*Missing*	20						
Lip competence							<0.005
Competent	1,480	97.1	1,429	96.6	51	3.5	
Incompetent	45	3.0	39	86.7	6	13.3	
*Missing*	21						
Canine occlusion							0.009
At least one canine Class II	261	18.1	243	93.1	18	6.9	
Both canines Class I	1,064	73.7	1.030	96.8	34	3.2	
At least one canine Class III (no canines Class II)	118	8.2	116	98.3	2	1.7	
*Missing*	103						

*Note*. Severe trauma: extensive fracture with pulp involvement, tooth displacement, necrotic/discolored tooth, total tooth loss due to trauma; *SD*: standard deviation.

**Figure 3:**
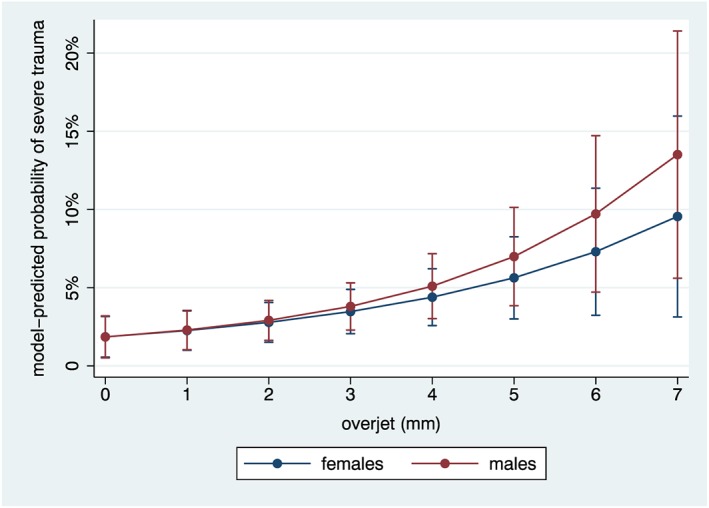
Final multivariable logistic regression model‐predicted probabilities and 95% confidence intervals of severe trauma, for males and females, according to overjet (mm)

The final multivariable model for severe trauma (extensive fracture with pulp involvement, tooth displacement, necrotic discolored tooth, or total tooth loss due to trauma) is presented in Table 3. The model included terms for children's age, sex, lip competence, and overjet. Overjet remained positively associated with severe trauma in multivariate analysis, *OR* = 1.40, 95% CI [1.23, 1.60], per added millimeter, corresponding to an absolute 1.3 p.p., 95% CI [0.7, 1.8], probability increase in the likelihood of severe trauma per millimeter of overjet. Figure 3 illustrates the multivariable model‐predicted probabilities of severe trauma according to overjet values ranging from 0 to 7 mm, stratified by sex. Overall, the model explained a small proportion (logistic model pseudo‐*R* ^2^ = 7.8%) of the observed variance in severe trauma. As such, it demonstrated weak predictive properties—it had 59% sensitivity and 70% specificity (area under the curve = 0.72), resulting in a 7.7% positive predictive value and 98% negative predictive value. The positive association between overjet and several dental trauma was replicated in categorical comparisons of “increased overjet” (i.e., >3 mm vs. ≤3 mm), *OR* = 3.83, 95% CI [1.99, 7.37], corresponding to an absolute 5.2 p.p., 95% CI [2.4, 8.0], probability increase for those with increased overjet (>3 mm). This model explained less variance as assessed by the logistic model's pseudo‐*R* ^2^ compared with the model for “continuous overjet” but correctly classified a higher proportion of participants (79% vs. 70%) and had higher positive predictive value (9.4% vs. 7.7%).

**Table 3:** Estimates of association (odds ratios [OR] and 95% confidence intervals [CI]) of demographic and clinical characteristics with the prevalence of severe dental trauma and corresponding predictive margins

Demographic or clinical characteristic	Association		Predicted marginal effect	
	*OR*	95% CI	Probability (percentage points)	95% CI
Model for continuous overjet				
Age (months)	1.02	[0.98, 1.05]	0.1	[0.0, 0.2]
Sex: male (referent: female)	1.10	[0.58, 2.10]	0.4	[−2.0, 2.8]
Lip: competent (referent: incompetent)	0.50	[0.15, 1.67]	−0.3	[−7.3, 2.0]
Overjet (mm)	1.40	[1.23, 1.60]	1.3	[0.7, 1.8]
Diagnostics: correctly classified = 70%; Se = 59%, Sp = 70%, PPV = 7.7%, NPV = 98%. Variance explained: logistic model pseudo‐*R* ^2^ = 7.8%				
Model for dichotomous “increased” overjet				
Age (months)	1.02	[0.98, 1.05]	0.7	[−0.1, 0.2]
Sex: male (referent: female)	1.09	[0.58, 2.06]	0.3	[−2.1, 2.8]
Lip: competent (referent: incompetent)	0.50	[0.15, 1.70]	−2.6	[−7.4, 2.1]
Overjet: “increased” (i.e., >3 mm vs. ≤ 3 mm)	3.83	[1.99, 7.37]	5.2	[2.4, 8.0]
Diagnostics: correctly classified = 79%; Se = 49%, Sp = 80%, PPV = 9.4%, NPV = 97%. Variance explained: logistic model pseudo‐*R* ^2^ = 5.7%				

*Note*. Se: sensitivity; Sp: specificity; PPV: positive predictive value; NPV: negative predictive value.

## 4 DISCUSSION

This study found a 47% overall prevalence of TDIs and 4% prevalence of severe TDIs among a community‐based sample of preschool‐age children. Community‐based oral health studies examining TDI are rare, especially in this young age group—this is an improvement over the study of clinical samples, comprising care‐seeking individuals who are more likely to have dental issues. Furthermore, few studies have investigated TDIs in the primary dentition, with only one known study in the United States (Jones et al., 1993). The 47% overall prevalence found in this study is slightly higher than previously reported estimates in the primary dentition. One potential explanation for the higher percentage of TDIs reported in this sample is that all children in this study were from low‐income families and were Medicaid‐eligible, as participation to EHS/HS is determined by qualification based on social and economic criteria. Some reports have shown that more children of lower socioeconomic status receive dental injuries compared with those in higher socioeconomic groups (Hamilton, Hill, & Holloway, 1997; Lalloo, 2003). Because simple crown fractures have minimal clinical consequences, this study considered severe trauma cases as the analytical outcome—extensive fractures with pulp involvement, tooth displacements, necrotic or discolored teeth, and total tooth loss due to trauma would require immediate management or intervention and thus were the ones most clinically relevant.

Several factors emerged as being associated with severe TDI; overjet and lip incompetence showed strong correlations, whereas age and canine occlusion showed weaker positive associations. In the final multivariate logistic regression model, age, sex, lip incompetence, and overjet were retained. This is consistent with numerous reports supporting the association between increased overjet and risk of TDIs to maxillary incisors in both the permanent and primary dentitions (Bastone et al., 2000; Bauss, 2008; Feldens et al., 2010; Goettems et al., 2012; Norton & O'Connell, 2012; Piovesan et al., 2012). Overbite, canine classification, and lip incompetence have also been linked to higher incidence of TDIs in the primary dentition (Bastone et al., 2000; Bonini et al., 2012; Goettems et al., 2012).

This study's results did not show a strong link between sex and incidence of severe trauma, consistent with other studies that suggest that there is no significant difference between sex and TDI in the primary dentition (Bastone et al., 2000; Bijella, Yared, Bijella, & Lopes, 1990; Onetto, Flores, & Garbarino, 1994). In the permanent dentition however, most studies report a higher percentage of dental trauma in males (Bastone et al., 2000; Borum & Andreasen, 2001). BMI, although not included in the final multivariate model, was weakly associated with increased incidence of TDI. Other reports examining postulated links between BMI to TDIs are also inconsistent. Soriano et al. found a statistically significant correlation between obesity and TDIs among a sample of 1,046 Brazilian children (Soriano et al., 2007). In contrast, Martins et al. (2014) found lower TDI prevalence (9%) among overweight/obese schoolchildren (BMI ≥ 85th percentile) ages seven to 14 years, compared with 13% TDI prevalence among schoolchildren who were not categorized as normal weight (15th < BMI < 85th percentile). Examining children's activity levels stratified by BMI might provide a better causal explanation for the postulated association between BMI and TDIs.

This study's findings should be framed by acknowledging its limitations. The clinical examiners were only calibrated on caries diagnosis and not on dental trauma detection, occlusion, overjet, and other intraoral and extraoral parameters. Another potential weakness is the assumption that tooth loss in certain circumstances was due to trauma instead of caries or incisal wear. It is not uncommon for children to have significant wear on the primary maxillary incisors. More severe forms of incisal wear that extend into dentin or expose the pulp are not as likely to be mistaken as trauma; however, there is less confidence in differentiating enamel‐only incisal wear and enamel‐only trauma. Evaluating tooth loss symmetry, caries risk, distribution of caries lesions, and number of teeth missing in the anterior region all aided in the clinical examiners' determination of the reason for tooth loss. Lastly, although the Frankl scale is a reliable rating system used frequently in pediatric dentistry to record the observed behavior and cooperation of the child in the clinical setting, it is not a comprehensive measurement of a child's risk‐taking behavior and may not be a good indicator of which children are more accident‐prone. Including information on activity level, participation in recreational activities or organized sports, and other behavioral markers, in a questionnaire could provide helpful information for a more complete picture of a child's risk for TDI.

In summary, including terms for behavioral factors, environmental factors, and oral factors into a risk model should provide parents, caregivers, dentists, and other health care professionals with a more contextual view of TDIs in order to reduce the prevalence of TDIs and identify those children at heightened risk of TDIs to provide proper education on prevention strategies. Additional studies, in larger community‐based samples including collection of additional possible predictors of dental trauma, are needed to further understand the interaction of factors that contribute to TDI in the primary dentition. This added information may enhance the education and communication opportunities between health care providers and caregivers and improve prevention strategies. Development of a risk assessment index as well as examination of the validity and generalization of a TDI risk index in external samples and populations is a logical future application of the current study.

After examination of behavioral, environmental, and oral factors, oral factors and particularly overjet proved to be the most significant predictors of TDI in this sample of preschool‐age children. Orthodontic interventions to reduce overjet, although advocated by some in the mixed dentition, would be focused more on interventions to eliminate nonnutritive sucking habits if present. Incorporating and operationalizing this information may help TDI prevention and related anticipatory guidance for families of preschool‐age children.

## 5 CONCLUSIONS

The following conclusions can be made based on this study's findings:

1. The prevalence of TDI among this community‐based sample of preschool‐age children was 47% and 8% of TDI cases were “severe,” defined as pulp exposure, tooth displacement, discolored or necrotic tooth, or tooth loss.
2. Overjet and lip incompetence were strong risk factors for TDIs in the primary dentition.
3. Accounting for age, sex, and lip incompetence, we found that each added millimeter of overjet was associated with 40% increased likelihood of severe dental trauma, corresponding to an absolute 1 p.p. approximate probability increase. Children with increased overjet (>3 mm) were 3.8 times as likely to have experienced severe TDI compared with those with ≤3 mm.

## WHY THIS PAPER IS IMPORTANT TO PEDIATRIC DENTISTS

- A clinically useful risk model for traumatic dental injuries helps pediatric dentists, pediatricians, and parents and caregivers quantitatively assess risk for children.
- A risk model or index for traumatic dental injuries, which includes terms for oral factors, BMI, sex, and behavioral and environmental factors, can be beneficial for family education, screenings, personalized prevention, risk reduction, and planning early orthodontic treatment or intervention.

## FUNDING INFORMATION

The ZOE 2.0 study is supported by National Institutes of Health/NIDCR grant #U01‐DE025046.

## CONFLICT OF INTEREST

None declared.

## References

[cre2165-bib-0001] American Academy of Pediatric Dentistry Clinical Affairs Committee – Behavior Management Subcommittee (2015). Guideline on behavior guidance for the pediatric dental patient. Reference Manual, 37(6), 180–193.

[cre2165-bib-0002] Andreasen, J. O. , & Ravn, J. J. (1972). Epidemiology of traumatic dental injuries to primary and permanent teeth in a Danish population sample. International Journal of Oral Surgery, 1, 235–239. 10.1016/S0300-9785(72)80042-5 4146883

[cre2165-bib-0003] Andreasen, J. O. , Sundström, B. , & Ravn, J. J. (1971). The effect of traumatic injuries to primary teeth on their permanent successors. Scandinavian Journal of Dental Research, 79, 219–283. 5286029 10.1111/j.1600-0722.1971.tb02013.x

[cre2165-bib-0004] Bastone, E. B. , Freer, T. J. , & McNamara, J. R. (2000). Epidemiology of dental trauma: A review of the literature. Australian Dental Journal, 45(1), 2–9. 10.1111/j.1834-7819.2000.tb00234.x 10846265

[cre2165-bib-0005] Bauss (2008). Influence of overjet and lip coverage on the prevalence and severity of incisor trauma. Journal of Orofacial Orthopedics, 69, 402–410. 10.1007/s00056-008-8805-1 19169637

[cre2165-bib-0006] Bijella, M. F. , Yared, F. N. , Bijella, V. T. , & Lopes, E. S. (1990). Occurrence of primary incisor traumatism in Brazilian children: A house‐by‐house survey. ASDC Journal of Dentistry for Children, 57(6), 424–427. 2258502

[cre2165-bib-0007] Bonini, G. C. , Bönecker, M. , Braga, M. M. , & Mendes, F. M. (2012). Combined effect of anterior malocclusion and inadequate lip coverage on dental trauma in primary teeth. Dental Traumatology, 28, 437–440. 10.1111/j.1600-9657.2012.01117.x 22364272

[cre2165-bib-0008] Born, C. D. , Divaris, K. , Hom, J. M. , & Rozier, R. G. (2015). Clinical predictors of traumatic dental injuries in preschool children. Journal of Dental Research, 94(Spec Iss A), 0435. (IADR/AADR)

[cre2165-bib-0009] Borum, M. K. , & Andreasen, J. O. (2001). Therapeutic and economic implications of traumatic dental injuries in Denmark: An estimate based on 7549 patients treated at a major trauma centre. International Journal of Paediatric Dentistry, 11, 249–258. 11570440 10.1046/j.1365-263x.2001.00277.x

[cre2165-bib-0010] Divaris, K. , & Joshi, A. (2018 Dec 19). The building blocks of precision oral health in early childhood: The ZOE 2.0 study. Journal of Public Health Dentistry. 10.1111/jphd.12303. PMC6584604 30566750

[cre2165-bib-0011] Ellis, R. G. , & Davey, E. W. (1970). Classification and treatment of injuries to the teeth of children (5th ed.). Chicago: Year Book Medical Publishers.

[cre2165-bib-0012] Fakhruddin, K. S. , Lawrence, H. P. , Kenny, D. J. , & Locker, D. (2008). Impact of treated and untreated dental injuries on the quality of life of Ontario school children. Dental Traumatology, 24, 309–313. 10.1111/j.1600-9657.2007.00547.x 18410390

[cre2165-bib-0013] Feldens, C. A. , Kramer, P. F. , Ferreira, S. H. , Spiguel, M. H. , & Marquezan, M. (2010). Exploring factors associated with traumatic dental injuries in preschool children: A Poisson regression analysis. Dental Traumatology, 26, 143–148. 10.1111/j.1600-9657.2009.00858.x 20070348

[cre2165-bib-0014] Feliciano, K. M. P. C. , & de França Caldas, A. Jr. (2006). A systematic review of the diagnostic classifications of traumatic dental injuries. Dental Traumatology, 22, 71–76. 10.1111/j.1600-9657.2006.00342.x 16499629

[cre2165-bib-0015] Ginnis, J. , Ferreira Zandoná, A. G. , Slade, G. D. , Cantrell, J. , Antonio, M. E. , Pahel, B. T. , … Divaris, K. (2019). Measurement of early childhood oral health for research purposes: Dental caries experience and developmental defects of the enamel in the primary dentition. Methods in Molecular Biology. In Press 10.1007/978-1-4939-9012-2_39 PMC6642073 30838597

[cre2165-bib-0016] Glendor, U. (2009). Aetiology and risk factors related to traumatic dental injuries—A review of the literature. Dental Traumatology, 25, 19–31. 10.1111/j.1600-9657.2008.00694.x 19208007

[cre2165-bib-0017] Goettems, M. L. , Azevedo, M. S. , Correa, M. B. , da Costa, C. T. , Wendt, F. P. , Schuch, H. S. , … Torriani, D. D. (2012). Dental trauma occurrence and occlusal characteristics in Brazilian preschool children. Pediatric Dentistry, 34, 104–107. 22583880

[cre2165-bib-0018] Goslee, M. T. , Lee, J. Y. , Rozier, R. G. , & Quinonez, R. B. (2006). The impact of dentoalveolar trauma on oral health‐related quality of life in young children and their families. Masters of Public Health Thesis. University of North Carolina‐Chapel Hill, Chapel Hill, NC.

[cre2165-bib-0019] Hamilton, F. A. , Hill, F. J. , & Holloway, P. J. (1997). An investigation of dentoalveolar trauma and its treatment in an adolescent population. Part 1: The prevalence and incidence of injuries and the extent and adequacy of treatment received. British Dental Journal, 182, 91–95. 10.1038/sj.bdj.4809313 9055474

[cre2165-bib-0020] Jones, M. L. , Mourino, A. P. , & Bowden, T. A. (1993). Evaluation of occlusion, trauma and dental anomalies in African‐American children of metropolitan Headstart programs. The Journal of Clinical Pediatric Dentistry, 18, 51–54. 8110614

[cre2165-bib-0021] Kaste, L. M. , Gift, H. C. , Bhat, M. , & Swango, P. A. (1996). Prevalence of incisor trauma in persons 6 to 50 years of age: United States, 1988‐1991. Journal of Dental Research, 75, 696–705. 10.1177/002203459607502S09 8594093

[cre2165-bib-0022] Lalloo, R. (2003). Risk factors for major injuries to the face and teeth. Dental Traumatology, 19, 12–14. 10.1034/j.1600-9657.2003.00139.x 12656849

[cre2165-bib-0023] Lee, J. Y. , & Divaris, K. (2009). Hidden consequences of dental trauma: The social and psychological effects. Pediatric Dentistry, 31, 96–101. 19455926

[cre2165-bib-0024] Lenzi, M. M. , Alexandria, A. K. , Ferreira, D. M. T. P. , & Maia, L. C. (2015). Does trauma to the primary dentition cause sequelae in permanent successors? A systematic review. Dental Traumatology, 31, 79–88. 10.1111/edt.12149 25382149

[cre2165-bib-0025] Martins, V. M. , Sousa, R. V. , Rocha, E. S. , Leite, R. B. , Gomes, M. C. , & Granville‐Garcia, A. F. (2014). Assessment of the association between overweight/obesity and traumatic dental injury among Brazilian schoolchildren. Acta Odontológica Latinoamericana, 27, 26–32. 25341257

[cre2165-bib-0026] Nguyen, P. M. , Kenny, D. J. , & Barrett, E. J. (2004). Socioeconomic burden of permanent incisor replantation on children and parents. Dental Traumatology, 20, 123–133. 10.1111/j.1600-4469.2004.00235.x 15144442

[cre2165-bib-0027] Norton, E. , & O'Connell, A. C. (2012). Traumatic dental injuries and their association with malocclusion in the primary dentition of Irish children. Dental Traumatology, 28, 81–86. 10.1111/j.1600-9657.2011.01032.x 21794080

[cre2165-bib-0028] Oliveira, L. B. , Marcenes, W. , Ardenghi, T. M. , Sheiham, A. , & Bonecker, M. (2007). Traumatic dental injuries and associated factors among Brazilian preschool children. Dental Traumatology, 23, 76–81. 10.1111/j.1600-9657.2005.00413.x 17367453

[cre2165-bib-0029] Onetto, J. E. , Flores, M. T. , & Garbarino, M. L. (1994). Dental trauma in children and adolescents in Valparaiso, Chile. Dental Traumatology, 10, 223–227. 10.1111/j.1600-9657.1994.tb00074.x 7843064

[cre2165-bib-0030] Pagadala, S. , & Tadikonda, D. C. (2015). An overview of classification of dental trauma. International Archives of Integrated Medicine, 2(9), 157–164.

[cre2165-bib-0031] Piovesan, C. , Guedes, R. S. , Casagrande, L. , & Ardenghi, T. M. (2012). Socioeconomic and clinical factors associated with traumatic dental injuries in Brazilian preschool children. Brazilian Oral Research, 26(5), 464–470. 10.1590/S1806-83242012000500014 23018232

[cre2165-bib-0032] Shulman, J. D. , & Peterson, J. (2004). The association between incisor trauma and occlusal characteristics in individuals 8‐50 years of age. Dental Traumatology, 20, 67–74. 10.1111/j.1600-4469.2004.00234.x 15025688

[cre2165-bib-0033] Soriano, E. P. , Caldas, A. F. Jr. , De Carvalho, M. V. , & Amorim Filho, H. A. (2007). Prevalence and risk factors related to traumatic dental injuries in Brazilian schoolchildren. Dental Traumatology, 23, 232–240. 10.1111/j.1600-9657.2005.00426.x 17635357

[cre2165-bib-0034] Wendt, F. P. , Torriani, D. D. , Assunção, M. C. F. , Romano, A. R. , Bonow, M. L. M. , da Costa, C. T. , … Hallal, P. C. (2010). Traumatic dental injuries in primary dentition: Epidemiological study among preschool children in South Brazil. Dental Traumatology, 26, 168–173. 10.1111/j.1600-9657.2009.00852.x 20089072

[cre2165-bib-0035] World Health Organization . Fact sheets. Available at: http://www.who.int/mediacentre/factsheets/fs318/en/. Accessed 2019‐1‐5.

[cre2165-bib-0036] Zaleckiene, V. , Peciuliene, V. , Brukiene, V. , & Drukteinis, S. (2014). Traumatic dental injuries: Etiology, prevalence and possible outcomes. Stomatologija, Baltic Dental and Maxillofacial Journal, 16, 7–14. 24824054

